# Basal Levels of Salivary Alpha-Amylase Are Associated with Preference for Foods High in Sugar and Anthropometric Markers of Cardiovascular Risk

**DOI:** 10.3390/bs8100094

**Published:** 2018-10-16

**Authors:** Ernesto Tarragon, Jakob Stein, Jobst Meyer

**Affiliations:** 1 Institute of Psychobiology, Department of Neurobehavioral Genetics, University of Trier, 54290 Trier, Germany; b.stein89@gmail.com (J.S.); meyerjo@uni-trier.de (J.M.); 2 Department of Psychobiology, Faculty of Health Sciences, University Jaume I, 12071 Castellon, Spain; 3 Department of Psychobiology and Methodology on Behavioral Sciences, Faculty of Psychology, Universidad Complutense de Madrid, 28223 Pozuelo de Alarcón, Spain

**Keywords:** salivary alpha-amylase, food preference, eating behaviour, body composition, glycaemic index, carbohydrates

## Abstract

Salivary alpha-amylase (sAA) influences the perception of taste and texture, features both relevant in acquiring food liking and, with time, food preference. However, no studies have yet investigated the relationship between basal activity levels of sAA and food preference. We collected saliva from 57 volunteers (63% women) who we assessed in terms of their preference for different food items. These items were grouped into four categories according to their nutritional properties: high in starch, high in sugar, high glycaemic index, and high glycaemic load. Anthropometric markers of cardiovascular risk were also calculated. Our findings suggest that sAA influences food preference and body composition in women. Regression analysis showed that basal sAA activity is inversely associated with subjective but not self-reported behavioural preference for foods high in sugar. Additionally, sAA and subjective preference are associated with anthropometric markers of cardiovascular risk. We believe that this pilot study points to this enzyme as an interesting candidate to consider among the physiological factors that modulate eating behaviour.

## 1. Introduction

Salivary alpha-amylase (sAA) hydrolases starch into smaller oligosaccharides as the first step of carbohydrate digestion. In addition, this enzyme is involved in the perception of taste [1,2] and texture [3]. This property is notably relevant in relation to eating, as physicochemical attributes determine the orosensory response (i.e., palatability) to food [4,5] and can therefore play a key role in modulating motivational (approach, consumption) and affective (hedonic response) constituents of eating behaviour [6]. Hence, together with psychological and cultural factors, physiology can be particularly important in establishing food preferences. Whether early physiological components of food processing, like salivary amylase activity, promote preference towards foods with relevant psychological salience is a query yet to be answered. 

Recently, a low copy number of the salivary amylase gene (*AMY1*) has been linked to a greater risk of developing obesity [7,8]. However, given that obesity is a multifactorial condition [9,10,11], how this genetic variation leads to an obese phenotype is still unclear. Foods rich in refined starch and sugar have been pointed out as major contributors to obesity and overeating [12,13,14,15]. Therefore, as the number of copies of *AMY1* is directly correlated with the amount of sAA [7], a reasonable question would be whether this enzyme influences the behavioural outcome related to weight gain, like the preference for foods with fattening properties. 

In the last decade, a trend pointing at higher carbohydrate intake as a determinant factor for obesity gained momentum. However, research has shown that it is not the carbohydrate content per se that is related to weight gain, but how processed these carbohydrates are and how fast they can increase blood glucose [16]. Based on this feature, foods are attributed a glycaemic index (GI), with a higher GI meaning a faster increasing glucose concentration in blood. There is evidence showing a clear relationship between GI and weight gain [17]. However, many researchers point to the glycaemic load (GL) instead of the GI as a more relevant measure, since GL takes into account the portion size and the carbohydrate content per portion. Regardless, both GI and GL have proved useful when exploring the relationship between obesity and carbohydrates [18,19]. Given the influence of sAA on orosensory properties, and how it affects later absorption of carbohydrates, it is reasonable to ponder how the GI and GL of different foods affect food preference.

In this pilot study, we explored two hypotheses to address this matter. First, given the relationship between *AMY1*, sAA, and obesity, we hypothesised that high levels of sAA would be associated with a low preference for refined carbohydrates. Here, we define “preference” as a construct with two dimensions, one subjective, and other self-reported behaviour. We believe this distinction is appropriate given that both constituents should be distinguished in the context of eating behaviour, since the desire to eat certain foods, or groups of food (subjective component), is not necessarily followed by actual consumption (behavioural component) [20]. Second, given the digestive properties of sAA, we expected lower levels of this enzyme to be associated with self-reported behavioural preference better than with subjective preference for these foods. In addition, because of the association between the intake of high GI foods and overweight, we also explored the relationship between body composition and basal levels of sAA. Finally, given the reported differences between men and women with regards to food preferences [21], we also tested sex differences.

## 2. Material and Methods

### 2.1. Participants and Procedure

Sixty participants (21 males and 39 females, from age 18 to 41) were recruited from the University of Trier via e-mail digest to fill in the preliminary version of the Food Craving Inventory in German (FCI-DE). Volunteers were invited to come to the laboratory facilities where the study would take place. Smoking, pregnancy, and previous or current history of psychiatric and cardiovascular disorders were criteria for exclusion. After a brief introduction, body measures from the participants were taken with a measurement tape ranging from 0 to 180 cm. Together with height and body weight, neck, forearm, wrist, waist, and hip circumferences were registered. These measures would be later used to estimate body composition features. After this, the FCI-DE first, and the German version of the Three-Factor Eating Questionnaire second, were handed to each participant. The order of the questionnaires was selected to avoid highlighting that restrained eating practices could affect responses to food craving measures. The total time to complete both questionnaires was 10 to 15 min. Upon completion, questionnaires were given back, and unstimulated saliva was collected. After this, participants received either 5 euros or a credit exchange sticker of a 30 min value. Informed consent was obtained from all individual participants included in the study. All procedures complied with the principles from the University Ethics Committee for Experimental Procedures with human subjects.

### 2.2. Body Composition

Height and waist circumference were registered in cm and weight was measured in kilograms (kg). Body Max Index (BMI) and Waist-to-Height Ratio (WHR) were calculated from body measures. Body Fat percentage (BF%) and Body Adiposity Index (BAI) were estimated using the standardised formulas developed by Deuremberg [22] and Bergman [23], respectively.

### 2.3. Evaluation of Food Preference

Taking the FCI as a starting point, we created a list of food items to evaluate both subjective and self-reported behaviour dimensions of food preference. The first part of the instrument (subjective scale) asked the participant how often, from 1 “never” to 5 “always/almost always”, he or she had felt cravings for a given food in the last week (Appendix A). The second part of the instrument (self-reported behaviour scale), asked, on the same item list, how often the person gave in to this appetence (Appendix B). The sum of the different scores of each scale conformed the Total Inventory Score (TIS), a measure used to evaluate the overall preference. A nutritional breakdown of the foods was performed, and the total carbohydrate, sugar, and starch content of each food was determined according to the tables of food composition [24] and the food database from the German government. The GI of the products was obtained from the University of Sydney database and the publication by Foster-Powell and collaborators [25]. The GL of each product was calculated as the result of the GI and total carbohydrate content product, divided by 100 [GL = (GI × total carbohydrate)/100)]. Items with GI ≥ 55 and GL ≥ 10 were considered “high” in the respective categories, according to generalised consensus. Mean values were considered to categorise foods as high/low in starch and high/low in sugar. Subjective and self-reported behaviour scores of each participant were calculated as the mean of all food items. Scale scores for the food categories (High/Low starch, sugar, GI, and GL) were calculated as the mean of the foods included in each category.

### 2.4. Salivary Alpha-Amylase Determination

Unstimulated saliva was collected between 8:30 a.m. and 3:30 p.m. Participants were instructed to come to the laboratory fasted (minimum 3 h). Salivette (Sarstedt, Nümbrecht, Germany) tubes were used to collect the saliva, following the manufacturer’s protocol. Briefly, the swab was removed from the Salivette and placed inside the mouth. After one minute of chewing, the swab was placed back inside its tube. Samples were stored until further analysis at 4 °C. For sAA determination, Salivettes were centrifuged at 4 °C, 10,000 rpm for 10 min. Alpha-amylase activity was determined by the standard ELISA procedure (Sigma-Aldrich Chemie Gmbh, Munich, Germany). 

### 2.5. Data Analysis

From the 60 volunteers, three (one man and two women) had to be excluded from the analysis because of extreme amylase levels and BMI values (women), as well as failure to complete the questionnaires (man). Normality of the data was checked with the skewness value and the Shapiro-Wilk test of normality. A *t*-test was performed to analyse differences between groups. Correlation and regression analyses were performed to explore the relationship between the variables. Cohen’s d and Cohen’s f^2^ were calculated for the effect size of group comparison and regression analysis, respectively [26]. Confident intervals (CI) are also reported. Participants were included in the healthy or overweight groups, depending on their WHR [27]. Given the ultradian variability of salivary amylase, the time at which the saliva samples were taken was also analysed. Data values are shown as mean ± S.E.M. Standard statistical significance was established at an alpha below 0.05. In those analysis where multiple comparisons were performed (*t*-test), a false discovery rate was applied following the Benjamini-Hochberg procedure. Amylase activity is shown as the log transformation of unit saliva per ml (U/mL). All the statistical procedures were conducted using the Statistical Software Package SPSS (version 23; IBM SPSS, Chicago, IL, USA).

## 3. Results

### 3.1. Participant Characteristics

Demographic and anthropometric data from the participants are presented in Table 1. Men presented a slight although non-significant overweight value (25.34 vs. overweight ≥25) in terms of BMI. In terms of BAI, WHR, and BF %, both sexes fell into the normal healthy range [23,28,29]. Basal levels of sAA were similar in both sexes. Men and women differ significantly in their BMI (*t* = −5.21, *p* < 0.001), BF% (*t* = 4.01, *p* < 0.001), WHR (*t* = −5.13, *p* < 0.001), and BAI (*t* = −5.14, *p* < 0.001).

### 3.2. Food Preference

The correlation between subjective and self-reported behavioural measures was strong and highly significant for TIS (r = 0.720, *p* < 0.001), foods high in starch (r = 0.669, *p* = 0.001), foods high in sugar (r = 0.630, *p* = 0.003), foods with high GI (r = 0.534, *p* = 0.015), and foods with high GL (r = 0.553, *p* = 0.011). 

**Figure 1:**
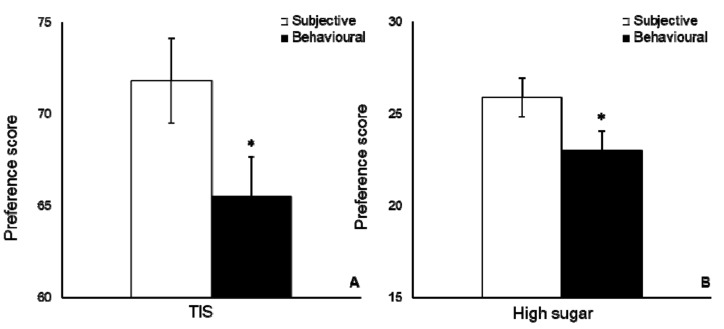

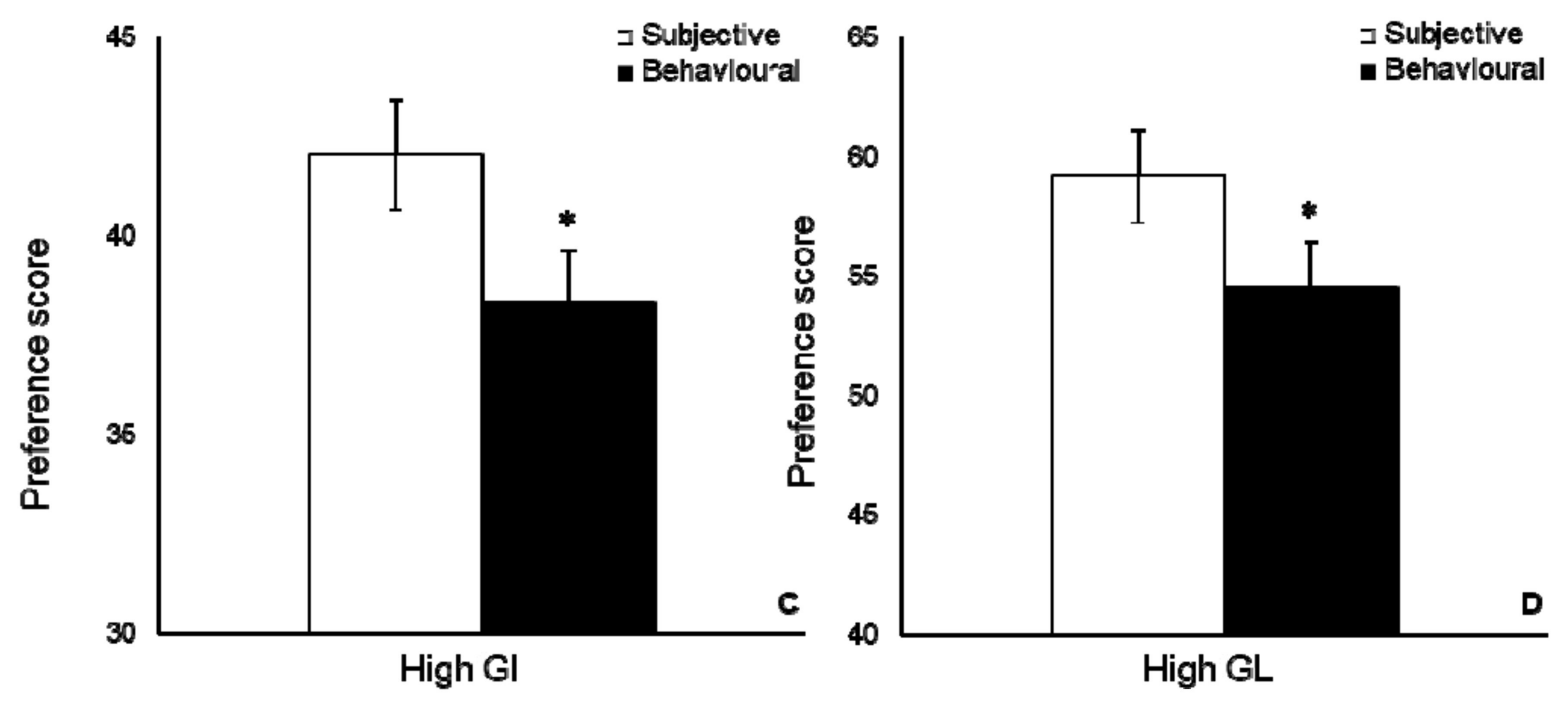
Subjective and self-reported behavioural preference. Preference score for Total Score Inventory (TIS) (**A**), foods high in sugar (**B**), foods with high GI (**C**), and foods with high GL (**D**). Behavioural = Self-reported behavioural. Data is presented as mean ± s.e.m. * Significant *p* < 0.01.

The *t*-test showed higher scores for subjective preference in comparison with self-reported behavioural preference in TIS (*p* < 0.001, d = 0.49, CI = 3.07 to 9.39), the high sugar category (*p* < 0.001, d = 0.488, CI = 1.53 to 5), the high GI category (*p* < 0.001, d = 0.528, CI = 2.03 to 6.57), and the high GL category (*p* < 0.001, d = 0.466, CI = 2.14 to 8.02) (Figure 1). We found no differences between men and women in terms of subjective and self-reported behavioural preference. 

### 3.3. Food Preference and Anthropometric Markers of Health Risk

A significant, positive correlation was found in women between BMI and self-reported behavioural preference TIS (r = 0.287, *p* = 0.038), foods high in sugar (r = 0.421, *p* = 0.004), with high GI (r = 0.305, *p* = 0.03), and with high GL (r = 0.295, *p* = 0.034). Furthermore, in women, BF% correlated with self-reported behavioural preference for foods high in sugar (r = 0.400, *p* = 0.006), with high GI (r = 0.286, *p* = 0.039), and with high GL (r = 0.280, *p* = 0.042). In men, self-reported behavioural preference for foods high in sugar correlated negatively with BMI (r = −0.421, *p* = 0.032) and BF% (r = −0.386, *p* = 0.046). No correlation was found between subjective preference and body measurements in men or in women.

**Figure 2:**
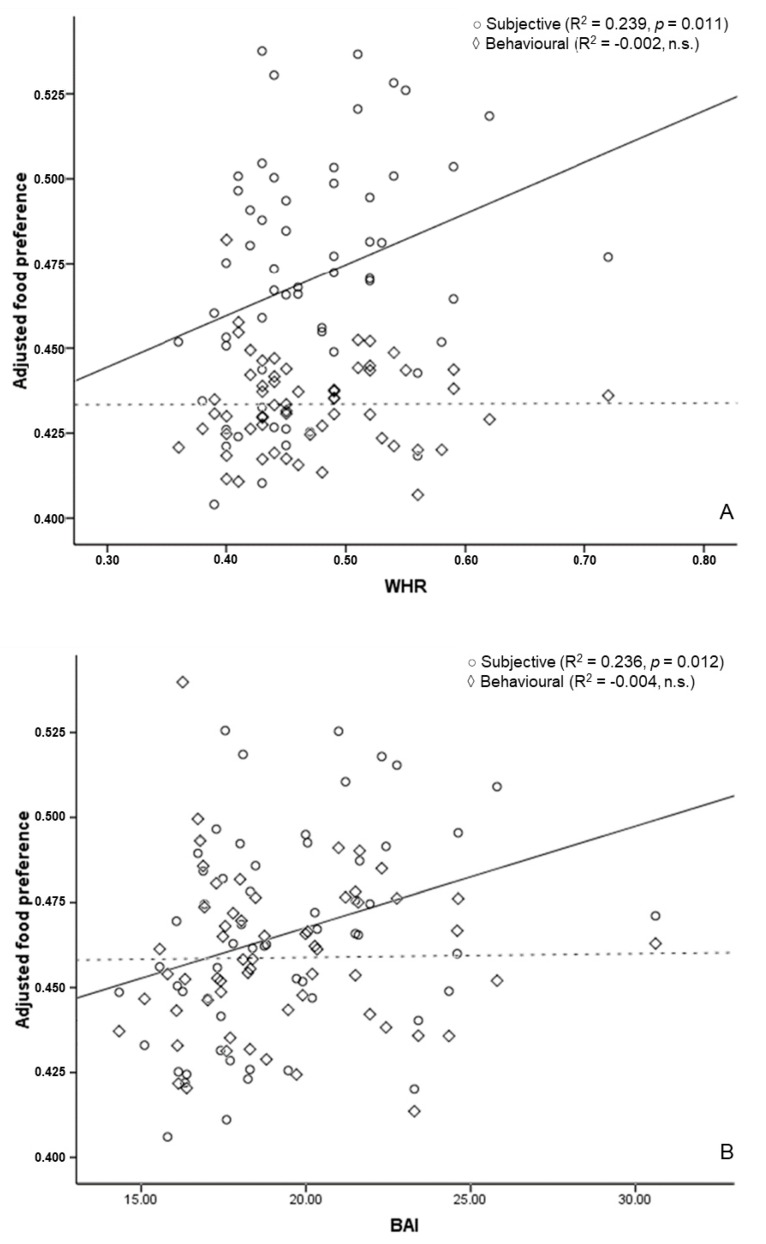
Food preference and anthropometric markers of cardiovascular risk in men and women. Adjusted predicted value of food preference for Waist-to-Hip Ratio (**A**) and Body Adiposity Index (**B**) in total sample. The straight line represents the line of best fit of the subjective category. The dotted line represents the line of best fit of the behavioural category.

The regression analysis showed a significant effect of subjective preference on WHR (R^2^ = 0.239, *p* = 0.011, f^2^ = 0.377) and BAI (R^2^ = 0.236, *p* = 0.012, f^2^ = 0.372) when men and women were analysed together. Self-reported behavioural preference had no impact on body measurements (Figure 2). When the analysis was performed on men and women separately, neither subjective nor self-reported behavioural preference seemed to be significantly associated with body measurements.

### 3.4. Effect of Time in sAA Levels

We performed a regression analysis to examine if the saliva samples were influenced by sAA levels. When the total sample was analysed, no effect was found. This was consistent when we analysed the samples by sex. Given the lack of influence of the moment in which the saliva was collected, further analysis was performed, omitting this variable.

### 3.5. sAA and Anthropometric Markers of Health Risk

A significant, positive correlation was found between sAA and WHR (r = 0.229, *p* = 0.039) and BAI (r = 0.231, *p* = 0.038) when data from both sexes were analysed together. When analysed separately, this correlation was only observed in women. No significant relationship was found in men (Table 2).

**Figure 3:**
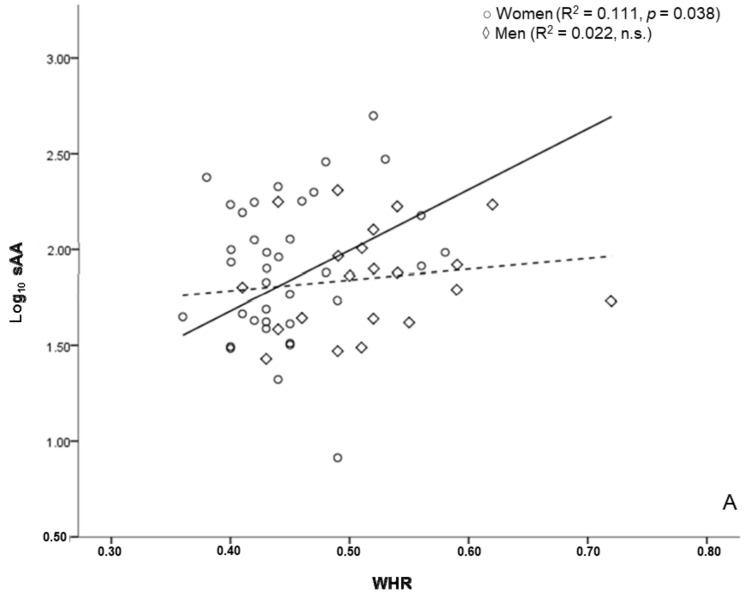

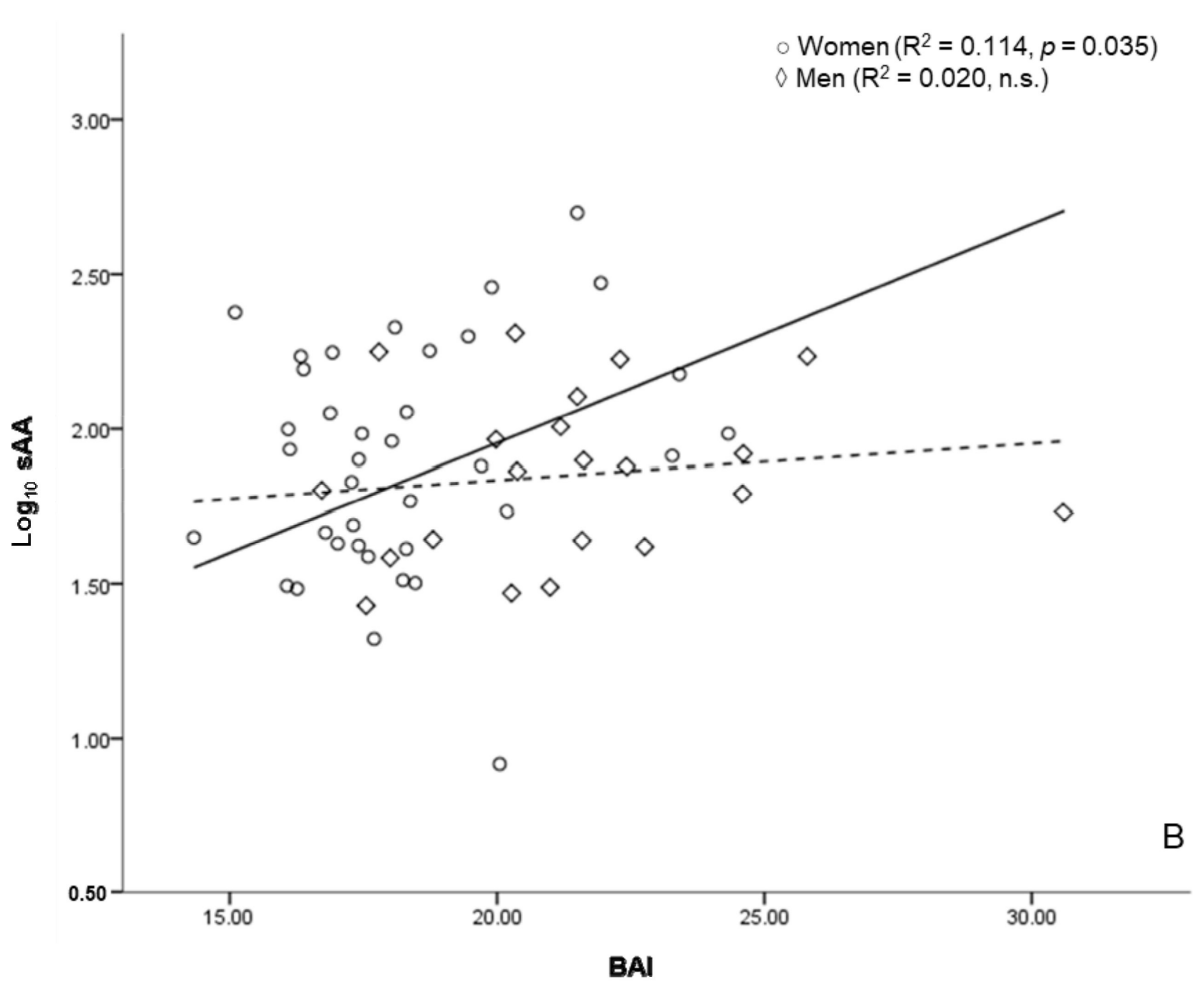
Salivary alpha-amylase and anthropometric markers of cardiovascular risk. Association of the log_10_ transformed sAA values of Waist-to-Hip Ratio (**A**) and Body Adiposity Index (**B**). The straight line represents the line of best fit of the subjective category. The dotted line represents the line of best fit of the behavioural category.

When the regression analysis was performed on men and women together, no association between sAA and body measurements was found. When split by sex, however, sAA was associated with WHR (R^2^ = 0.111, *p* = 0.038, f^2^ = 0.142) and BAI (R^2^ = 0.114, *p* = 0.035, f^2^ = 0.128) in women (Figure 3). No association was found in men.

### 3.6. sAA and Food Preference

A significant, negative correlation was found between sAA and subjective preference for foods high in sugar (r = −0.244, *p* = 0.034) when data from men and women were analysed together. When split by sex, the correlation analysis showed a significant, negative relationship between sAA and preference for food high in sugar, both in subjective (r = −0.323, *p* = 0.026) and self-reported behavioural measures (r = −0.283, *p* = 0.045). No correlation was found in men (Table 3). 

**Figure 4:**
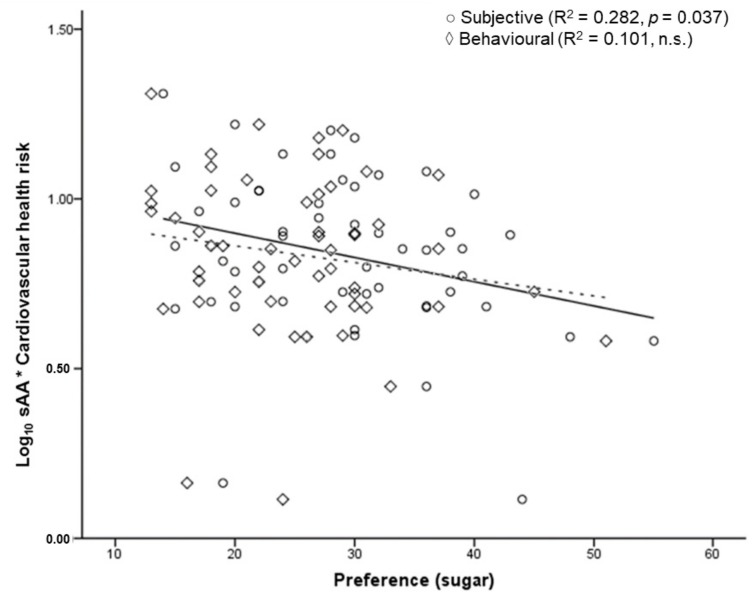
Salivary alpha-amylase and food preference. Association between the log_10_ transformed sAA values and WHR in terms of subjective preference for foods high in sugar (in women). The straight line represents the line of best fit of the subjective category. The dotted line represents the line of best fit of the behavioural category.

The regression analysis indicated no relation between sAA and food preference in the overall sample. A second analysis was performed, including WHR and BAI in the model. When these anthropometric markers of health risk were accounted for, sAA was inversely associated with subjective, but not self-reported behavioural preference for foods high in sugar (R^2^ = 0.282, *p* = 0.037, f^2^ = 0.095). Although not as significant, this result was consistent when the analysis was performed for women only (R^2^ = 0.104, *p* = 0.051, f^2^ = 0.126) (Figure 4). No effect was observed for men.

### 3.7. SAA, Body Composition, and Eating Behaviour

A correlation analysis was performed to explore the relationship between the three dimensions of the TFEQ and sAA first, and body composition, secondly. No correlation was found between the restrained eating scale of the TFEQ and sAA, BMI, WHR, BAI, or BF%. 

## 4. Discussion

We put together a list of foods to assess in two dimensions, according to personal preference. Subjective and self-reported behavioural preference scales reflected how often the person had the desire to eat a specific food, and how often they would give into that appetence, respectively. Eating is not only a matter of re-stabilising physiological homoeostasis. Therefore, distinguishing these two domains of preference may be relevant. In this work, we explored the hypothesis of basal levels of sAA being associated with food preference and various body measurements associated with health status, coherent with the evidence presented in recent research [7,30,31]. In addition, we explored whether food preference, measured as subjective and behavioural dimensions (self-reported), would affect body composition in different ways. Our intention was to provide preliminary evidence of the possible association between these factors. Nevertheless, given the reported differences between men and women in food preference, we also looked at this in our study.

Several conclusions can be drawn from our data. First, food preference can be effectively differentiated into separate dimensions; one subjective, characterised by volition without the need of an action towards consuming food; and another in which this will is followed by consummatory behaviour. Further, our data indicate that, although being highly correlated, each of these dimensions are differentially associated with (1) basal levels of sAA, (2) various food categories, (3) body composition measurements, and (4) sex. Such results are consistent with other research reporting differences between subjective and self-reported behavioural evaluation of the same foods [32,33]. 

Second, the subjective, but not the behavioural, dimension seems to effectively predict body composition; specifically, WHR and BAI. Interestingly, both subjective and self-reported behavioural preference correlate with body composition. We found that this relationship is different in men and women, up to a point where self-related behavioural preference is negatively associated with these variables in men. Concretely, our data show a negative association between BMI and BF% and self-reported behavioural preference for foods high in sugar (in men). This sex-related discrepancy could be explained by previously reported differences describing how men tend to prefer more savoury rather than sweet foods. Assuming that foods with a greater sugar content are associated with a greater measure of obesity, it is expected that lowering their intake would correlate with better body composition values. 

Finally, we demonstrate that sAA is associated with body composition in women only and that this enzyme affects subjective, but not self-reported behavioural, food preference. Concretely, we show that basal levels of fasting sAA inversely predict subjective preference for foods high in sugar, but not other food groups or behavioural measures. 

Understanding the relationship between sAA and the two preference dimensions becomes pertinent after considering the evidence showing that lower levels of sAA represent a greater risk of developing insulin tolerance and type 2 diabetes [31]. Societies and individuals with a lower copy number of the amylase gene present a greater risk for developing obesity, regardless of environmental and other biological factors [7]. Interestingly, the amount of amylase found in saliva correlates directly with the number of copies of the *AMY1* gene [7]. Again, this is consistent with our findings of lower sAA, reflecting fewer *AMY1* copies, associated with a greater preference for foods that have been similarly linked to weight gain and demonstrated fattening properties [34,35]. Coherently, our results indicate that lower levels of sAA correlate positively with greater preference, mostly for foods high in sugar. This suggests that lower levels of this enzyme could be an important aspect of the physiological environment by which individuals are influenced when making dietary choices. We think this is particularly relevant, given that it could contribute to partially explain the obesity phenotype in those individuals carrying low copy numbers of the amylase gene. 

The second hypothesis of this study was that sAA could also be related to some extent to body composition measures associated with cardiovascular health, such as WHR. Contrarily to what we expected, the analysis revealed a significant and positive association between sAA and two indicators of cardiovascular health in adults: body adiposity and WHR [36,37,38]. However, according to the hypothesis of amylase, this relationship should be inverse.

Some studies suggest that greater sAA activity indicates a better adaptation to starchy foods, as carbohydrates can be more rapidly digested and blood glucose more easily regulated [30,39]. This opens the door to considering this enzyme as an indicator of lacking specific nutrients, maybe acting as a motivational trigger towards foods high in such missing components. This is consistent with the observation of food preference and health factors. Since lower levels of sAA would increase (subjective) preference for certain foods, the probability of consuming them (behaviour) rises, finally contributing to the intensification of such factors. 

It is also important to note various other limitations in this study. First, in relation to the absent relationship between sAA and self-reported behavioural preference, this could be explained by participants exerting some kind of restrained eating strategy [20,40]. Interestingly, self-reported behavioural preference did not correlate with the cognitive control measure from the FEV, although it did positively correlate with the disinhibition subscale (data not shown). Another reason for this subjective/behavioural discrepancy could be palatability (more palatable foods may present better chance to be approached), or the opportunity to consume a particular food. However, this seems unlikely given that all the categories presented highly palatable and available food items. 

Second, regarding body composition, we acknowledge the deficiencies of methods based on mathematical formulas compared with more precise techniques. Although we found a positive relationship between sAA and body composition, the utility of these procedures is still a matter of debate, with some studies showing no difference between BMI and other anthropometric measures [41]. On the other hand, previous research has shown that these WHR and BAI efficiently predict the risk of developing obesity-related conditions. Segheto and collaborators demonstrated that in the absence of more sophisticated methods (i.e., DEXA scan), BAI is an effective estimator of adiposity [42]. In addition, WHR has proved a better predictor of dyslipidaemia, hypertension, and diabetes, when compared with other anthropometric measures, like BMI [43]. In addition, regardless of the adequacy of the methods used, most of the anthropometric values measured fell within a range considered healthy. Therefore, the clinical relevance of this finding is unclear at best. Regarding this, studies in a clinical population could help to better elucidate our findings.

Finally, we did not take into consideration the phase of the menstrual cycle. A higher appetence for foods high in fat and sugars has been reported during the menstrual cycle [44]. Unfortunately, we did not register information on the participants’ cycle phase. Nevertheless, it is very unlikely that this factor would make a difference in our results, given the reduced chance that all women, during different days, were under the same cycle by the time they participated in the study. Also, although the time of the day was considered, the ultradian variations in amylase levels were not introduced as a confounding variable in the analysis. We also acknowledge this as a limitation. However, considering that the time variable did not show a significant effect, we believe that for the purposes of this pilot study, it is not a major issue. Nevertheless, we reckon that future investigations should not neglect this variable in the design.

## 5. Conclusions

To the best of our knowledge, this is the first study exploring the relationship between sAA and food preference, understood as a motivational construct. Our findings suggest that sAA could influence eating behaviour towards consuming certain food groups (i.e., high in sugar), and body composition, particularly in women. However, given the limitations mentioned before, conclusions should be taken carefully. Regardless of this, we believe that the results presented here point to factors worth considering further. Taking basal levels of sAA as a biomarker associated with relevant health measures could be a promising tool to consider when approaching eating behaviour, whether it is in a clinical or research context. Therefore, we trust that this work reports interesting results on the relationship between psychophysiological aspects of eating behaviour and could be of use in future studies that further explore this relationship between salivary enzymes and food preference.

## Figures and Tables

**Table 1:** Anthropometric measures and sAA in men and women.

	Men	Women
	(n = 21)	(n = 37)
	Mean ± s.d., Asymmetry ± d.e.	
Weight	82.62 ± 12.69, 0.95 ± 0.501	62.58 ± 13.16, 0.13 ± 0.388
Body Mass Index	25.34 ± 3.62, 1.52 ± 0.501	21.94 ± 4.73, 0.49 ± 0.388
Body fat %	20.03 ± 5.40, 0.93 ± 0.501	25.23 ± 5.13, 1.01 ± 0.388
Body Adiposity Index	21.43 ± 3.18, 1.12 ± 0.501	18.07 ± 2.27, 1.05 ± 0.388
Waist to Hip Ratio	0.52 ± 0.07, 1.06 ± 0.501	0.44 ± 0.05, 1.04 ± 0.388
sAA	85.06 ± 11.80, 1.01 ± 0.501	95.85 ± 12.50, 1.09 ± 0.388

sAA = salivary alpha-amylase, s.d. = standard deviation, d.e. = deviation error.

**Table 2:** Correlation between salivary alpha-amylase and anthropometric measurements.

		Log_10_ sAA
Women	BMI	0.154
	WHR	0.323 *
	BAI	0.327 *
	BF %	0.209
Men	BMI	0.119
	WHR	0.149
	BAI	0.14
	BF %	0.173

BMI = Body Mass Index, WHR = Waist-to-Hip Ratio, BAI = Body Adiposity Index, BF = Body Fat. * Significant, *p* < 0.05.

**Table 3:** Correlation between salivary alpha-amylase and food categories.

Sex	Preference	Category	Log_10_ sAA
Women	Subjective	TIS	−0.254
		Starch	−0.115
		Sugar	−0.323 *
		GI	−0.244
		GL	−0.258
	Self-reported behaviour	TIS	−0.206
		Starch	−0.081
		Sugar	−0.283 *
		GI	−0.154
		GL	−0.173
Men	Subjective	TIS	−0.072
		Starch	0.105
		Sugar	−0.138
		GI	0.046
		GL	−0.044
	Self-reported behaviour	TIS	0.044
		Starch	0.241
		Sugar	−0.011
		GI	0.209
		GL	0.125

TIS = Total Inventory Score, GI = Glycaemic Index, GL = Glycaemic Load. * Significant, *p* < 0.05.

## Appendix A. Food Item Inventory. Subjective Scale

*“**Craving**” ist ein Fachbegriff aus der Psychologie, der das nahezu unbezwingbare Verlangen einer Person um schreibt, ein bestimmtes Nahrungsmittel zu sich zu nehmen.*

Bitte markieren Sie, wie oft Sie im letzten Monat “*Craving*” auf die folgenden Lebensmittel gefühlt haben.

**Table A1:** List of foods and frequency to which they are craved.

	Nie	Selten	Manchmal	Häufig	Immer/Fast Immer
Backfisch	□	□	□	□	□
Frühstücksspeck (*Bacon*)	□	□	□	□	□
Belegte Brötchen	□	□	□	□	□
Brezeln	□	□	□	□	□
Brötchen	□	□	□	□	□
*Brownies*	□	□	□	□	□
Chips	□	□	□	□	□
Cracker	□	□	□	□	□
Döner	□	□	□	□	□
Donuts	□	□	□	□	□
Eiscreme	□	□	□	□	□
Frikadellen	□	□	□	□	□
Gesalzene Erdnüsse	□	□	□	□	□
Haferflocken	□	□	□	□	□
Hamburger	□	□	□	□	□
Honig	□	□	□	□	□
Kekse	□	□	□	□	□
Kuchen	□	□	□	□	□
Marzipan	□	□	□	□	□
Müsli	□	□	□	□	□
Nudeln	□	□	□	□	□
Nussnougatcreme	□	□	□	□	□
Ofenkartoffeln	□	□	□	□	□
Pfannkuchen	□	□	□	□	□
Pizza	□	□	□	□	□
Pommes frites	□	□	□	□	□
Reis	□	□	□	□	□
Schokolade, Pralinen	□	□	□	□	□
Steak	□	□	□	□	□
Süße Backwaren	□	□	□	□	□
Süßigkeiten	□	□	□	□	□
Toastbrot	□	□	□	□	□
Waffeln	□	□	□	□	□
Wurst	□	□	□	□	□

## Appendix B. Food Item Inventory. Behavioural Scale

*Am diese Mal im letzten Monat wenn Sie auf ein besonderes Lebensmittel “Craving” gefühlt haben, wie oft haben Sie nachgegeben und aßen?*

**Table A2:** List of foods and frequency to which cravings are given in.

	Nie	Selten	Manchmal	Häufig	Immer/Fast Immer
Backfisch	□	□	□	□	□
Bacon (Frühstücksspeck)	□	□	□	□	□
Belegte Brötchen	□	□	□	□	□
Brezel	□	□	□	□	□
Brötchen	□	□	□	□	□
*Brownies*	□	□	□	□	□
Chips	□	□	□	□	□
Cracker	□	□	□	□	□
Döner	□	□	□	□	□
Donuts	□	□	□	□	□
Eiscreme	□	□	□	□	□
Frikadellen	□	□	□	□	□
Gesalzene Erdnüsse	□	□	□	□	□
Haferflocken	□	□	□	□	□
Hamburger	□	□	□	□	□
Honig	□	□	□	□	□
Keks	□	□	□	□	□
Kuchen	□	□	□	□	□
Marzipan	□	□	□	□	□
Müsli	□	□	□	□	□
Nudeln	□	□	□	□	□
Nussnougatcreme	□	□	□	□	□
Ofenkartoffeln	□	□	□	□	□
Pfannkuchen	□	□	□	□	□
Pizza	□	□	□	□	□
Pommes frites	□	□	□	□	□
Reis	□	□	□	□	□
Schokolade, Pralinen	□	□	□	□	□
Steak	□	□	□	□	□
Süße Backwaren	□	□	□	□	□
Süßigkeiten	□	□	□	□	□
Toastbrot	□	□	□	□	□
Waffel	□	□	□	□	□
Wurst	□	□	□	□	□
